# Reliability in Standardization of Iron(III) and Titanium(III) Solutions in Volumetric Analysis

**DOI:** 10.1021/acsomega.1c03074

**Published:** 2021-08-05

**Authors:** Toshiaki Asakai, Toshihiro Suzuki

**Affiliations:** National Institute of Advanced Industrial Science and Technology, National Metrology Institute of Japan, 1-1-1 Umezono, Tsukuba, Ibaraki 305-8563, Japan

## Abstract

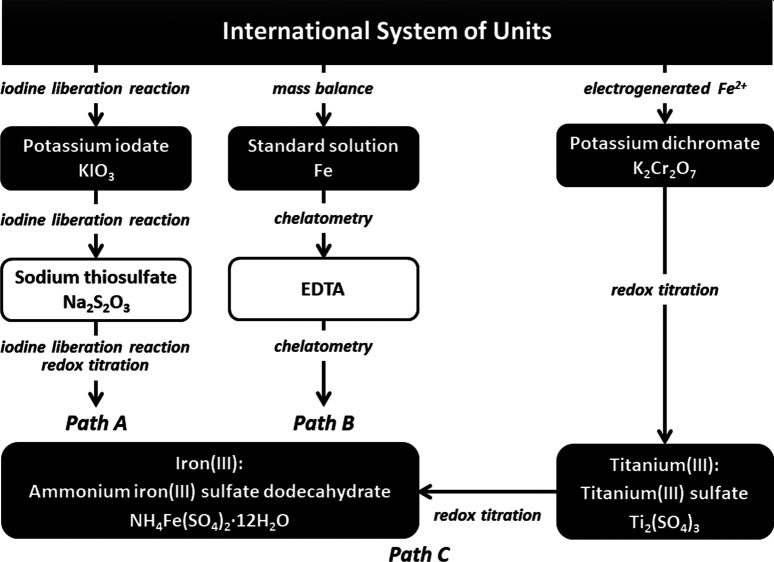

Titanium(III) is a useful strong reductant and is usually standardized with iron(III) in volumetric analysis. Iron(III) is widely used as an oxidant and is usually standardized with thiosulfate ions through an iodine liberation reaction. The evaluation of the standardization procedure for iron(III) with thiosulfate ions is therefore essential to ensure the reliability of standardized titanium(III) solutions. To investigate the titration procedure for iron(III), two different titrations were performed: redox titration with thiosulfate ions through an iodine liberation reaction and chelatometric titration with disodium dihydrogen ethylenediaminetetraacetate. Subsequently, for the investigation of standardization of iron(III), titanium(III) was assayed through two titration paths: redox titration with standardized iron(III) and redox titration with standard potassium dichromate. The reliability of titrimetric procedures was evaluated by applying several different stoichiometric reactions to each chemical. All titrimetric procedures were consistent with each other within their expanded uncertainties and were capable of providing reliable volumetric standards with careful operations presented in this study.

## Introduction

A reliable titanium(III) standard solution became necessary when the authors were trying to determine the purity of the perchlorate salt.^1^ Perchlorate explosively reacts with organic materials; in contrast, the stoichiometric reduction of perchlorate is more difficult in a series of oxyanions of chlorine due to its inactivity.^2,3^ A highly strong reductant, that is, titanium(III), is needed to stoichiometrically reduce perchlorate. Titanium(III) is often standardized with iron(III).^4^ Iron(III) is usually standardized with thiosulfate through an iodine liberation reaction.^5,6^ Thiosulfate is usually standardized with a certified reference material (CRM) of iodate or dichromate. These titrimetric methods play a key role in the accuracy of the titration results.^7^ Useful metrological information on the reliability of the volumetric standards such as these very weak oxidant and very strong reductant has not been found. The aim of the present study is to evaluate the reliability of the titrimetric procedures for iron(III) and titanium(III) as a volumetric standard.

Ammonium iron(III) sulfate dodecahydrate is often used as a source material for the reagent solution or the standard solution of iron(III).^5^ A standardization procedure for iron(III) is as follows: iodine (triiodide ions) liberated by iron(III) in an acidic potassium iodide solution (eq 1) is titrated with a sodium thiosulfate solution (eqs 2 and 3); the sodium thiosulfate solution is standardized with standard potassium iodate or potassium dichromate (eqs 2 and 3):
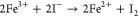
1
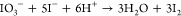
2
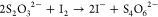
3

The iodine liberation process is significantly affected by the amounts of acids and potassium iodide, the waiting time for liberation, and light. The process, therefore, plays a key role in the accuracy of standardization of iron(III). One of the authors has discussed several appropriate standardization procedures through the iodine liberation reaction for strong oxidants such as iodate, cerium(IV), bromate, periodate, dichromate, and osmium(VIII).^8−13^ The accuracy of the titrimetric procedure for iron(III) would be lower than that for these strong oxidants because the lower oxidizing ability of iron(III) would lead to a smaller oxidation rate of iodide ions. Longer experimental time would lead to larger biases due to side reactions such as the oxidation of iodide ions by atmospheric oxygen and the volatilization of generated iodine.

**Figure 1:**
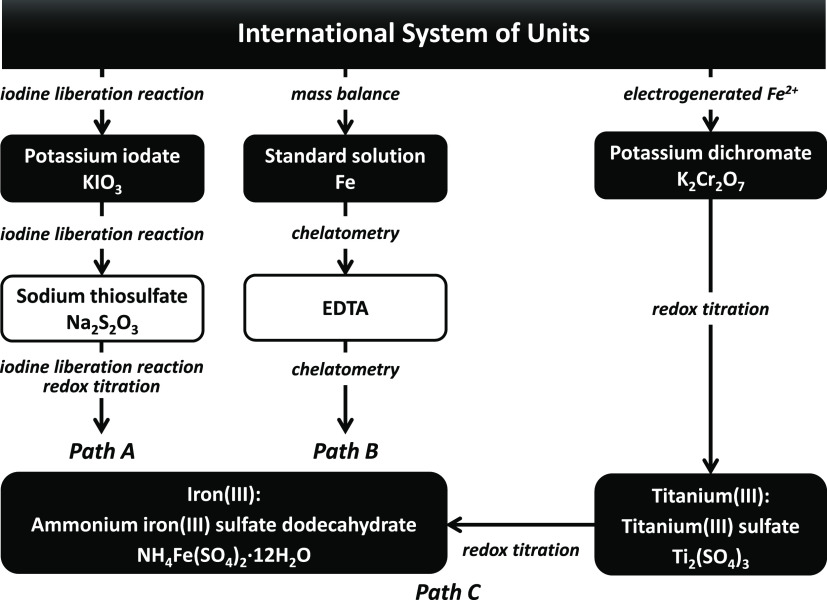
Experimental design for iron(III) and titanium(III) measurements.

In the present study, iron(III) was assayed through two titration paths to investigate the iodine liberation reaction (Figure 1): redox titration with thiosulfate through the iodine liberation reaction and chelatometric titration with disodium dihydrogen ethylenediaminetetraacetate (EDTA). The concentration of the thiosulfate ion solution was standardized with standard potassium iodate; the EDTA solution was standardized with the Japanese national standard solution of iron.

Titanium(III) sulfate [Ti_2_(SO_4_)_3_] or titanium(III) chloride [TiCl_3_] are often used as a source material of titanium(III). The sulfate salt was chosen as a source material of titanium(III) in this study to avoid the oxidation of chloride ions in redox titrations. Titanium(III) was assayed through two titration paths to examine the accuracy of titration procedures (Figure 1): redox titration with standardized iron(III) (eq 4) and redox titration with standard potassium dichromate (eq 5).^14,15^
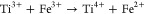
4
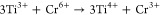
5

The reliability of titrimetric procedures for iron(III) and titanium(III) solutions was discussed by performing several different titrations based on CRMs, the certified values of which were traceable to the International System of Units (SI).

## Results and Discussion

### Assay of Iron(III) with EDTA

An approximately 1000 mg kg^–1^ solution as Fe of ammonium iron(III) sulfate dodecahydrate was titrated with an EDTA solution, which was standardized with a national Fe standard solution.

The titrimetric result of the mass fraction of Fe in the solution was 994.943 mg kg^–1^. Assuming that the purity of ammonium iron(III) sulfate dodecahydrate as the source material was 100%, the calculated mass fraction of the gravimetrically prepared solution was 999.950 mg kg^–1^ as Fe. Consequently, the purity (mass fraction) of ammonium iron(III) sulfate dodecahydrate in solid was 99.50% (RSD 0.0034%, *n* = 5, “RSD” means “experimental relative standard deviation,” and *n* is the number of measurements under a repeating condition).

### Assay of Iron(III) with the Thiosulfate–Iodine Liberation Reaction Examined by Constant Voltage Biamperometry

**Figure 2:**
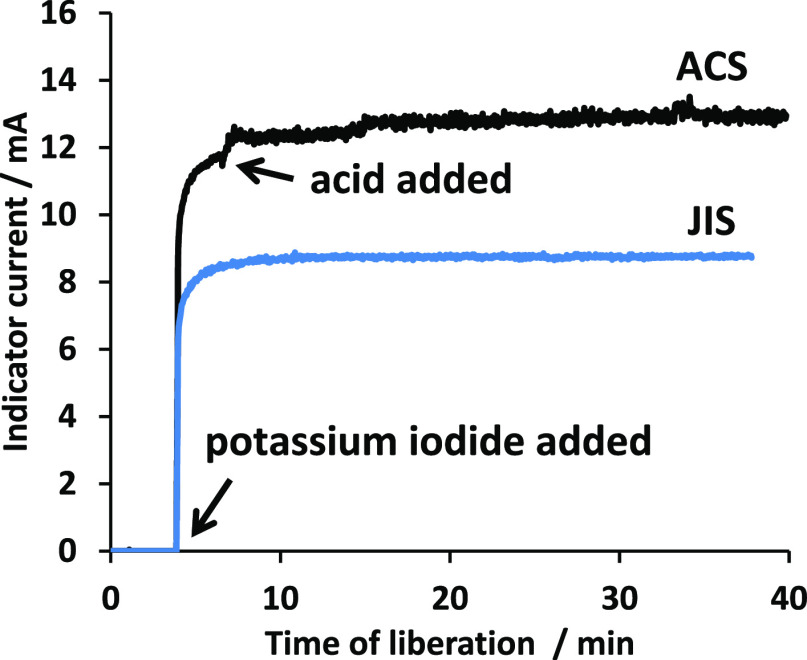
Profile of iodine liberation by oxidizing iodide ions with iron(III). The iodine liberation process was monitored by constant voltage biamperometry (applied constant voltage of 500 mV). The indicator currents were recorded after adding 3 g of potassium iodide.

A profile of iodine liberation monitored by constant voltage biamperometry is shown in Figure 2. This liberation condition was a similar one to that specified in American Chemical Society (ACS).

The pH of the solution of 1.8 g of ammonium iron(III) sulfate dodecahydrate dissolved in 50 mL of pure water was about 2. Iodine was promptly liberated after adding 3 g of potassium iodide because the solution was already acidic. The liberation seemed to be slightly accelerated by adding 6 mL of 6 mol L^–1^ hydrochloric acid.

The iodine liberation was apparently slower than that driven by potassium iodate.^8^ The liberation was almost completed within a few seconds in the case of using potassium iodate; the liberation by iron(III) would roughly take more than 5 or 10 min.

The slightly modified liberation conditions specified in the Japanese Industrial Standard (JIS) were as follows: 1 g of ammonium iron(III) sulfate dodecahydrate, 3 g of potassium iodide, and 13.2 mL of 6 mol L^–1^ hydrochloric acid. The sample size was smaller, and the amount of acid in JIS was larger than that of ACS.^5,6^ The liberation profile in JIS monitored by biamperometry was similar to that in ACS.

### Assay of Iron(III) with the Thiosulfate–Iodine Liberation Reaction Examined by Gravimetric Titration with Thiosulfate Ions

**Figure 3:**
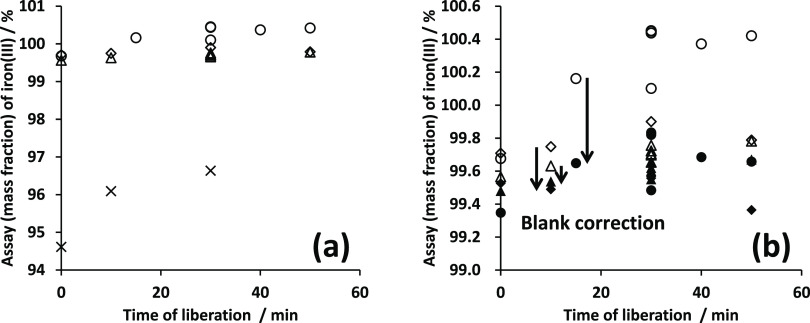
Dependency of assays of ammonium iron(III) sulfate dodecahydrate on the time of iodine liberation. Panel (b) is an enlarged view of panel (a) and is displayed with blank corrections. Open circle indicates 1 g of sample, 3 g of potassium iodide, and 13.2 mL of 6 mol L^–1^ hydrochloric acid; open triangle (uncorrected) and solid triangle (corrected) indicate 1.8 g of sample, 3 g of potassium iodide, and 6 mL of 6 mol L^–1^ hydrochloric acid; open diamond (uncorrected) and solid diamond (corrected) indicate 1.8 g of sample, 3 g of potassium iodide, and 13.2 mL of 6 mol L^–1^ hydrochloric acid; cross symbol indicates 1.8 g of sample and 3 g of potassium iodide without acid.

Dependencies of assay results of ammonium iron(III) sulfate dodecahydrate on the time of iodine liberation are shown in Figure 3a.

In the case without an acid, the assay results were apparently lower by 4 to 5% than the others regardless of the time of the iodine liberation. These lower results would be caused by incomplete iodine liberation since biamperometric investigations also indicated similar lower results before the addition of the acid (Figure 2). In the case with hydrochloric acid, these results were within a range of 99.5 to 100.5%.

**Figure 4:**
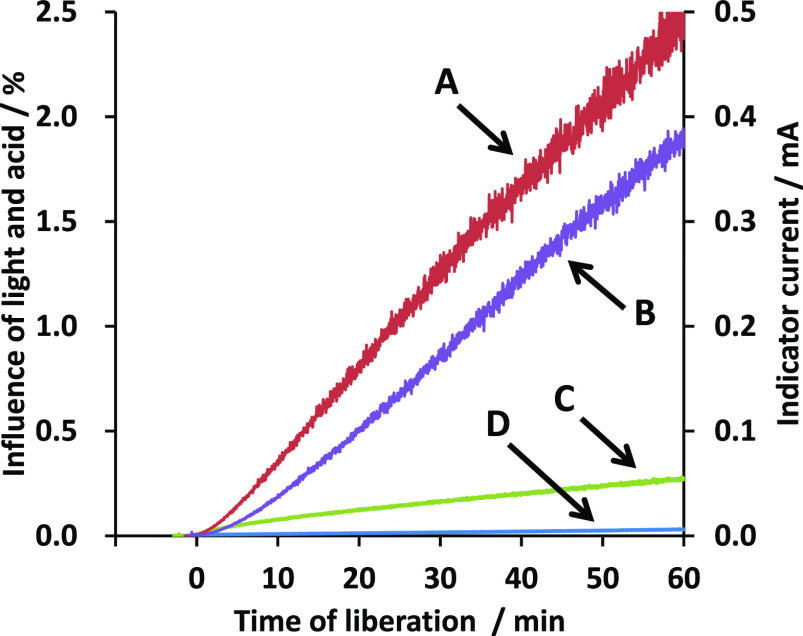
Impact of light and acid on the assays of 1.8 g of ammonium iron(III) sulfate dodecahydrate obtained using the biamperometric approach. Increases in background current after adding 3 g of potassium iodide and different amounts of acid without ammonium iron(III) sulfate dodecahydrate were monitored in a bright or dark room. (A) 13.2 mL of 6 mol L^–1^ hydrochloric acid, bright room; (B) 6 mL of 6 mol L^–1^ hydrochloric acid, bright room; (C) 13.2 mL of 6 mol L^–1^ hydrochloric acid, dark room; and (D) 6 mL of 6 mol L^–1^ hydrochloric acid, dark room.

Uncorrected data obtained using ACS and JIS conditions given in Figure 3b were inconsistent with each other and slightly had a dependency on time. The reason was air oxidation of iodide ions during the iodine liberation. Figure 4 shows changes in currents resulting from air oxidation of iodide ions. Using constant voltage biamperometry to detect liberated iodine, the influence of air oxidation of iodide ions under several conditions and its contribution to the assays for 1.8 g of ammonium iron(III) sulfate dodecahydrate were determined. The impact on the assays was larger with larger amounts of acid used under similar conditions. The impact on the assays was significantly larger in a bright room than that in a dark room. The presence of light could accelerate air oxidation of iodide ions in an acidic medium.

Figure 3b shows the results corrected with the bias caused by air oxidation of iodide ions for each assay. The results obtained under different conditions were much closer. Correction data in a bright room were used for the time of gravimetric titrations (6 min) and those in a dark room were used for the time of waiting for liberation (0 to 50 min) (see also Figure 4).

In summary, the assay results (mass fraction) of ammonium iron(III) sulfate dodecahydrate were 99.604% (RSD 0.038%, *n* = 5) in accordance with ACS results and 99.743% (RSD 0.173%, *n* = 4) in accordance with JIS results. The results obtained in accordance with JIS showed much bias caused by air oxidation of iodide ions and required larger correction. They showed larger deviation compared to ACS’s results. The major reason was much air oxidation of iodide ions due to higher acidic conditions.

### Brief Summary for Iron(III) Assays and Measurement Uncertainties

A summary of the measurement uncertainties for iron(III) through three titration paths is given in Table 1. The uncertainty of the molar mass of the standards was included in each certified value. The uncertainties of the molar mass of the sample iron(III) and buoyancy corrections for all chemicals were small enough in comparison with those of the other sources and were not combined.

**Table 1:** Uncertainty Budget for the Measurements of Iron(III)

uncertainty source	relative standard uncertainty, %		
	path A (thiosulfate)	path B (EDTA)	path C (Ti)
standard (potassium iodate, iron, and potassium dichromate)	0.011	0.050	0.008
repeatability against the standard	0.0023	0.0041	0.168
repeatability against the sample	0.038	0.0034	0.114
weighing of the standard	0.0013	0.0003	0.0009
weighing of titrants	0.0005	0.0010	0.0005
weighing of the sample	0.0011	0.0003	0.0008
			
combined standard uncertainty	0.040	0.050	0.20
expanded uncertainty (*k* = 2)	0.080	0.10	0.40

The assay result of ammonium iron(III) sulfate dodecahydrate titrated with an SI-traceable EDTA solution was 99.50% ± 0.10% (*k* = 2) (path B). The assay of the same sample using the iodine liberation reaction with an SI-traceable thiosulfate ion solution was 99.604% ± 0.080% (*k* = 2) (path A). One of the simplest tests, *E*_n_, was applied to the results obtained from paths A and B. The evaluation of *E*_n_ is often used to test the difference between the reference value and a reported value in proficiency testing^16^
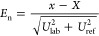
6where *E*_n_ is the *E*_n_ value, *x* is the reported value, *X* is the reference value, *U*_lab_ is the expanded uncertainty from a participant, and *U*_ref_ is the expanded uncertainty of the reference value. Inputting the results from paths A and B, the calculated *E*_n_ value was 0.81. This value was less than 1, and thus it indicated that the results were overlapped within their expanded uncertainties. Both titration paths therefore were available to accurately obtain the mass fraction of ammonium iron(III) sulfate dodecahydrate. In the route through the iodine liberation reaction, the use of a stopper beaker, an appropriate amount of acid, a quick titrating operation, and shielding from light were necessary.

### Assay of Iron(III) with Titanium(III)

An approximately 250 mol kg^–1^ solution of titanium(III) sulfate was titrated with standard potassium dichromate and ammonium iron(III) sulfate dodecahydrate standardized with EDTA.

The concentration of the titanium(III) solution measured was 269.76 mmol kg^–1^ (SD 0.45 mmol kg^–1^, *n* = 3, and “SD” means “experimental standard deviation”). Smaller number of measurements and larger SD were caused by the instability of titanium(III).

**Figure 5:**
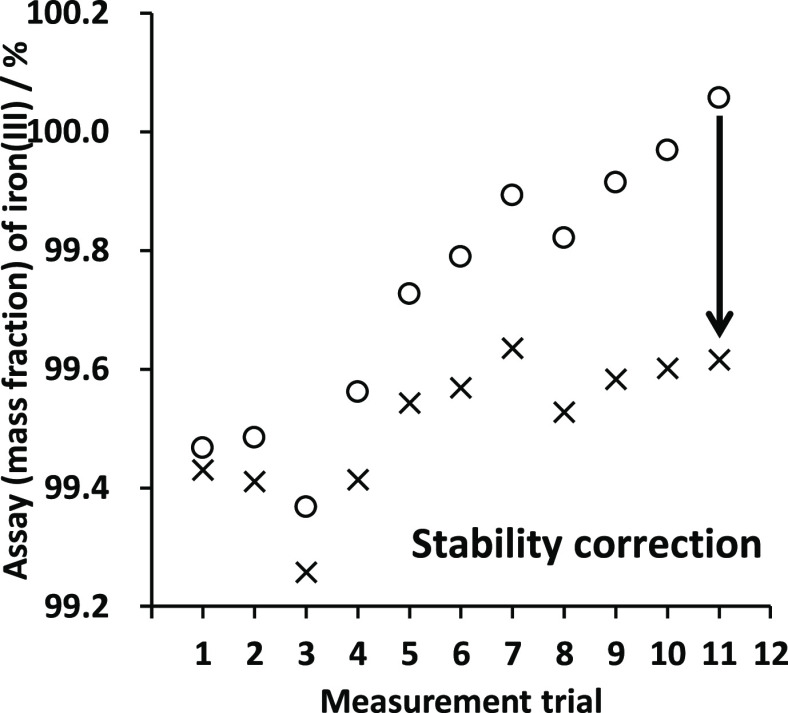
Assays of ammonium iron(III) sulfate dodecahydrate titrated with titanium(III) sulfate standardized with standard potassium dichromate. Open circle indicates uncorrected values and cross symbol indicates corrected values.

The results of the assay of ammonium iron(III) sulfate dodecahydrate calculated based on the concentration of titanium(III), 269.76 mmol kg^–1^, are shown in Figure 5. The assay results of ammonium iron(III) sulfate dodecahydrate increased with the order of the measurements. The time of the measurements was about 2.3 h. Judging from the standardization results with potassium dichromate, the concentration of titanium(III) decreased by 0.16% per hour during the measurements. The uncorrected results shown in Figure 5 were corrected using the concentrations of titanium(III) standardized with standard potassium dichromate before and after the measurement trials for iron(III) with titanium(III). By correcting the data using the instability of titanium(III), better assay results of iron(III) were obtained. Consequently, the assay result of ammonium iron(III) sulfate dodecahydrate titrated with titanium(III) based on standard potassium dichromate was 99.52% ± 0.40% (*k* = 2) (see Table 1). This expanded uncertainty was overlapped with the other titration paths. The results of all three titration paths therefore were obviously consistent with each other without calculating the *E*_n_ values.

## Conclusions

The reliability of titrimetric procedures for iron(III), titanium(III), and thiosulfate ions was determined by performing several different titrations based on CRMs, the certified values of which were traceable to the SI. Volumetric standard solutions of iron(III) and titanium(III) were evaluated through several different stoichiometric reactions: (a) redox reaction between iron(III) and thiosulfate via an iodine liberation reaction, (b) chelate-forming reaction between iron(III) and EDTA, and (c) redox reaction between iron(III) and titanium(III). In path A, the rate of iodine liberation by iron(III) was lower than that by other strong oxidants. Larger amounts of acids and iodide ions were needed to accelerate the reaction. Longer times for iodine liberation were required to complete the reaction. These reaction conditions lead to much oxidation of iodide ions by light and resulted in larger assay results of iron(III). The use of a stopper beaker during iodine liberation, smaller and appropriate amounts of acid, quick titrating operation, and shielding from light are necessary. Blank corrections are strongly recommended. In path B, the procedure showed better measurement repeatabilities. The issues when using this titration path are that a national Fe standard solution is not available in the market and the uncertainty of the standard solution is significantly larger than that of the measurement repeatability. In path C, the biggest issue is the instability of titanium(III) solutions although the reaction is feasible. Titanium(III) should be standardized as needed and promptly used. All titrimetric procedures were consistent with each other within their expanded uncertainties and were capable of providing reliable volumetric standards using the presented procedures. The information presented in the study has contributed to perform reliable titrimetry and allowed all analysts to obtain reliable results in chemical analyses.

## Experimental Section

### Chemicals

Ammonium iron(III) sulfate dodecahydrate is an analytical reagent-grade chemical specified in the JIS and was obtained from FUJIFILM Wako Pure Chemical Corporation, Osaka, Japan.^6^ Potassium iodate as a standard was NMIJ CRM 3006-a. This CRM was certified by coulometric titration based on Faraday’s laws of electrolysis, and its certified value was traceable to the SI. The certified value of the potassium iodate oxidant was 99.973% ± 0.022% (coverage factor *k* = 2, which gives a level of confidence of approximately 95%). A monoelemental standard solution of iron was NMIJ CRM 3611-a, which was used as a national standard. This CRM was certified by the mass balance method, and its certified value was traceable to the SI. The certified value of the mass fraction of iron was 992.9 mg kg^–1^ ± 1.0 mg kg^–1^ (*k* = 2).

Titanium(III) sulfate solution (20% in 1 to 4% sulfuric acid) was obtained from Alfa Aesar, MA, USA. Potassium dichromate as a standard was NMIJ CRM 3002-a. This CRM was certified by coulometric titration, and its certified value was traceable to the SI. The certified value of the potassium dichromate oxidant was 99.974% ± 0.015% (*k* = 2).

An EDTA titrant solution was prepared by dissolving 3.72 g of ethylenediaminetetraacetic acid disodium salt dihydrate (Dojindo Laboratories, Japan) in 1 L of Milli-Q water.

Analytical reagent-grade chemicals were used unless otherwise stated. Buoyancy corrections were always applied.^17,18^ The molar mass and the density of ammonium iron(III) sulfate dodecahydrate used were 482.198 g mol^–1^ and 1.71 g cm^–3^, respectively.^19,20^ The density of each solution was assumed to be 1 g cm^–3^ for their buoyancy corrections.

### Apparatus

Volumetric titration using a monoelemental national standard solution of iron, EDTA, and ammonium iron(III) sulfate dodecahydrate was performed with an automatic chelatometric titrator AT-420 of Kyoto Electronics Manufacturing Co., Ltd., Kyoto, Japan. Gravimetric titrations were carried out in other titration paths with a plastic syringe with a perfluoroalkoxy alkane needle and type XP26 and XP205 balances obtained from Mettler Toledo, Tokyo, Japan.

Spectrophotometry, constant voltage biamperometry, and potentiometry were applied to the end point detections for chelatometric titration, iodometric titration, and other redox titrations, respectively: a PTA-510 spectrophotometry adapter and a P-114 optical fiber dip-type sensor of Kyoto Electronics Manufacturing Co., Ltd., Kyoto, Japan for spectrophotometry; a type 7651 DC source and a type 7562 digital multimeter of Yokogawa Electric Corporation, Tokyo, Japan equipped with a dual-platinum electrode for biamperometry; and a type HM-30R potentiometer (pH meter) with a Pt–Ag/AgCl combination electrode of DKK-TOA Corporation, Tokyo, Japan for potentiometry were employed.

### Experimental Procedure—Iron(III) with EDTA and Thiosulfate

An approximately 1000 mg kg^–1^ solution as Fe was gravimetrically prepared by dissolving ammonium iron(III) sulfate dodecahydrate in pure water and kept in a high density, narrow-neck polyethylene (HDPE) bottle with an inner lid.

Approximately 10 g of iron solution was placed in a beaker, and 50 mL of pure water and an excess amount (ca. 20 mL) of ca. 0.01 mol L^–1^ EDTA were added. The solution pH was adjusted from 2.0 to 2.2 with diluted aqueous ammonia. The excess EDTA was back-titrated with ca. 0.01 mol L^–1^ bismuth nitrate using xylenol orange as an indicator. A blank test was carried out to calibrate the concentration ratio between the EDTA solution and the bismuth titrant. Ammonium iron(III) sulfate dodecahydrate as a sample solution and a monoelemental standard solution of iron were titrated in this manner. The mass fraction of ammonium iron(III) sulfate dodecahydrate as Fe was calculated by comparing both the titration results.

Approximately 1.8 g of ammonium iron(III) sulfate dodecahydrate was placed in a 100 mL tall beaker and dissolved in 50 mL of pure water. Approximately 3 g of potassium iodide and 6 mL of 6 mol L^–1^ hydrochloric acid were added to the solution. The liberation of iodine started after adding potassium iodide and the acid. This condition was similar to the one specified in ACS.^5^

Constant voltage biamperometry was utilized to roughly investigate the iodine liberation profile. Biamperometry is a method for monitoring the current between typically twin platinum electrodes where a constant voltage is applied (500 mV). The current flows in the presence of both iodide ions and triiodide ions through the reaction [3I^–^ ⇄ I_3_^–^ + 2e^–^] on each surface.^21^ The current was proportional to a certain amount of liberated iodine.

The purity of ammonium iron(III) sulfate dodecahydrate as Fe through the iodine liberation reaction was examined by gravimetric titration. Approximately 1.8 g of ammonium iron(III) sulfate dodecahydrate was placed in a 50 mL beaker and dissolved in 50 mL of pure water. Approximately 3 g of potassium iodide and 0 to 13.2 mL of 6 mol L^–1^ hydrochloric acid were added to the solution. The solution was titrated with a sodium thiosulfate solution at 0 to 50 min after the iodine liberation started. A stopper beaker was used during the liberation to prevent the vaporization of iodine. The concentration of the sodium thiosulfate solution was standardized with standard potassium iodate in advance. The end point was detected by constant voltage biamperometry. The indicator current proportionally decreased with the titration proceeding near the end point and the current was kept at zero amperes after the end point. The end point was the intersection between the linear regression curve of indicator currents versus the amounts of the sodium thiosulfate solution used and the baseline (0 μA).^22^

### Experimental Procedure–Iron(III) with Titanium(III)

An approximately 250 mol kg^–1^ solution of titanium(III) sulfate was prepared by diluting a titanium(III) sulfate solution (20% in 1 to 4% sulfuric acid) two times with 2 mol L^–1^ sulfuric acid. The solution was kept in a HDPE bottle of Nalgene.

Approximately 0.18 g of standard potassium dichromate was placed in a 50 mL beaker and dissolved with 20 mL of 2 mol L^–1^ sulfuric acid. The potassium dichromate solution was gravimetrically titrated with the titanium(III) solution on heating around 90 °C. The end point was detected by potentiometry with a Pt–Ag/AgCl combination electrode. The inflection point calculated by third-order polynomial approximation was decided as the end point.^22^

Approximately 1.6 g of ammonium iron(III) sulfate dodecahydrate was placed in a 50 mL beaker and dissolved with 20 mL of 2 mol L^–1^ sulfuric acid. The iron(III) solution was gravimetrically titrated with the titanium(III) solution on heating around 50 °C. The end point was detected in the same manner mentioned above.
